# Enhanced Mechanical Properties of Yellow ZrN Ceramic with Addition of Solid Solution of TiN

**DOI:** 10.3390/ma15217866

**Published:** 2022-11-07

**Authors:** Zongpeng Wu, Zhen Gao, Jun Zhao, Saisai Li, Qi Hao, Songlin Ran

**Affiliations:** 1 Engineering Trainning and Innovation Education Center, Anhui University of Technology, Maanshan 243002, China; 2 Gemmological Institute, Guangzhou City University of Technology, Guangzhou 510800, China; 3 School of Materials Science and Engineering, Anhui University of Technology, Maanshan 243002, China

**Keywords:** ZrN, TiN, solid solution, microstructure, mechanical properties

## Abstract

As a superhard ceramic with a yellow color and excellent electrical conductivity, ZrN has potential applications in the field of decoration, but it is limited by its poor mechanical properties. In this work, the mechanical properties of ZrN ceramic were improved by forming a (Zr, Ti)N solid solution via spark plasma sintering of a ZrN and TiN powder mixture. The influences of the amount of TiN additive on the sinterability, microstructure, color, and mechanical properties of ZrN ceramic were investigated. X-ray diffraction analysis, energy-dispersive spectroscopy, and microstructural images indicated that Ti atoms dissolved into a ZrN lattice, and a (Zr, Ti)N solid solution was formed during the sintering process. When the content of TiN was 10 vol%, the obtained (Zr, Ti)N composite exhibited the best comprehensive mechanical properties; the Vickers hardness, flexural strength, and fracture toughness were 15.17 GPa, 520 MPa, and 6.03 MPa·m^1/2^, respectively. The color coordinates and color temperature diagram revealed the addition of TiN hardly impacted the color performance of the ZrN ceramic.

## 1. Introduction

ZrN ceramic has excellent comprehensive properties, such as high hardness, excellent corrosion resistance, high thermal conductivity, excellent electrical conductivity, and wear resistance [1,2,3]. Therefore, ZrN ceramic is widely used in structural ceramics, protective coating, and high-temperature refractories. In addition, ZrN exhibits a yellow color, which can be used for parts of watches or other wear-resistant decorative products. However, the poor mechanical properties of monolithic ZrN ceramic restrict its further application.

The addition of the second phase is an effective way to overcome the drawbacks of ZrN ceramic. Tang et al. [4] investigated the influence of Zr and Ti additives on the densification process of hot-pressed ZrN ceramics. The results indicate that Zr and Ti additives can both facilitate the densification process. Liu et al. [5] reported the role of a ZrO_2_–Y_2_O_3_ sintering additive in the ZrN–ZrO_2_–Y_2_O_3_ system. Petukhov et al. [6] obtained ZrN–ZrB_2_ via spark plasma sintering (SPS) of ZrH_2_, BN, and B powder mixtures. It was reported that ZrB_2_ content significantly influenced the sinterability of ZrN. Zgalat-Lozynskyy et al. [7] fabricated ZrN–Si_3_N_4_ and ZrN–Si_3_N_4_–TiN composites via spark plasma sintering (SPS). They found that the relative density and Vickers hardness of the samples were improved when TiN was used as a sintering additive. Titanium nitride (TiN) demonstrated outstanding mechanical properties, such as high hardness, excellent chemical stability, and a high melting point [8,9]. Additionally, ZrN and TiN crystallize in the face-centered cubic (fcc) structure, resulting in the formation of solid solutions during the sintering process [10]. Numerous studies have proved that the solid solution can effectively improve the sintering performance and mechanical properties of composites [11,12,13,14]. Therefore, the introduction of TiN particles in the ZrN matrix would be an effective way to promote densification and improve mechanical properties.

In addition, the high melting point, strong covalent bonding, and low self-diffusion coefficients of ZrN and TiN make densification difficult. Therefore, conventional sintering methods such as pressureless sintering and hot pressing require higher temperatures and longer holding times to obtain dense composites. However, grains will grow during the sintering process, which will damage the performance of the composites. SPS is well-known for its use in the preparation of high-melting temperature ceramics with poor sinterability, offering the advantages of a faster heating rate, shorter dwell time, and limited grain growth [15,16,17]. Therefore, the SPS technique is a promising method for fabricating (Zr, Ti)N composites with high density and excellent properties. However, to date, there are no studies in the literature on (Zr, Ti)N composites sintered by SPS.

In this work, (Zr, Ti)N composites were fabricated via the SPS technique, and the influence of the amount of TiN additive on the sinterability, microstructure, color, and mechanical properties was studied. The phase compositions of the composites were studied by X-ray diffraction (XRD). The microstructures and chemical compositions of the composites were performed by field emission scanning electron microscopy (FESEM) with energy-dispersive spectroscopy (EDS). The mechanical properties of the composites, including Vickers hardness, flexural strength, and fracture toughness, were reported.

## 2. Materials and Methods

ZrN powder (mean particle size: 1~3 µm, Shanghai Naiou Nano Technology Co., Ltd., Shanghai, China) and TiN powder (mean particle size: 0.5 µm, Shanghai Naiou Nano Technology Co., Ltd., Shanghai, China) were used as starting materials. ZrN and TiN powders were mixed according to the formula ZrN–x vol% TiN with x = 0, 10, 20, and 30. The final products were denoted as ZN, ZT10, ZT20, and ZT30 according to the TiN content. The powders were mixed using a zirconia ball-milled in polyethylene bottles for 24 h with absolute ethanol as the ball-milling media. Then, the slurries were evaporated in a rotary evaporator at 60 °C (RE-52AA, Shanghai Yarong Biochemical Instrument Factory, Shanghai, China) and placed in a drying oven (DHG-9070A, Shanghai Bluepard Instrument Co., Ltd., Shanghai, China) at 90 °C for 24 h. After that, the mixtures were separately poured into a graphite mold, sintering in a vacuum at 2000 °C for 15 min under 50 MPa using the spark plasma sintering apparatus (SPS, SPS-20T-10, Shanghai Chen Hua Science and Technology Co., Ltd., Shanghai, China).

The density and porosity of the samples were measured using the Archimedes principle with deionized water as the immersion medium according to ASTM standard B311 [18]. The crystal phases and lattice parameters of obtained composites were examined using X-ray diffraction (XRD, Ultima IV, Rigaku, Japan). The microstructure was characterized using field emission scanning electron microscopy (FESEM, MIRA3 XMU, TESCAN, Czechia). The chromaticity coordinates of as-prepared samples were obtained using a fluorescence spectrometer (EX-1000, Everfine Photo-E-Info Co., Ltd., Hangzhou, China) with a 450 W xenon lamp as the excitation source (slit width 2 nm, Δλ = 2) and CIE chromaticity coordinate software. Vickers hardness and fracture toughness were determined using a hardness tester (HVS, Shanghai Shangcai Testermachine Co., Ltd., Shanghai, China) with a 19.6 N load for 15 s. Fracture toughness was determined using the formula derived by Evans et al. [19]. At least ten values were taken, and average values were used for each sample. The samples were processed by wire cut electrical discharge machining (WEDM, DK77, Ningbo Zhongyuan Machine Tool Co., Ltd., Ningbo, China) with dimensions of 2 mm × 3 mm × 25 mm (thickness, width, and length, respectively). The flexural strength of the samples was measured using an electric universal testing machine (AGS-X, Shimadzu Instruments Manufacturing Co., Ltd., Suzhou, China) with a crosshead speed of 0.5 mm/min and a span of 20 mm according to the three-point bending method. The flexural strength value for one sample was the average of the best five values.

## 3. Results

### 3.1. Phase Compositions

**Figure 1:**
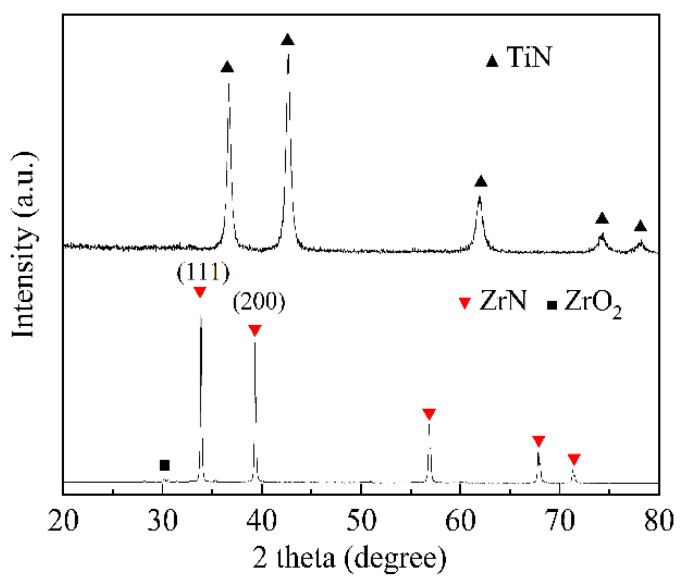
XRD patterns of raw ZrN powders and TiN powders.

**Figure 2:**
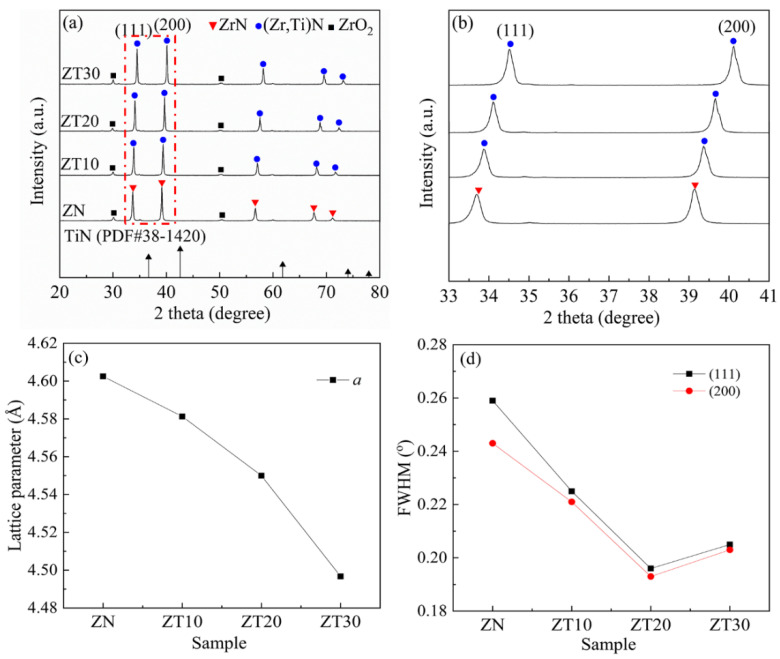
(**a**) XRD patterns of the sintered samples: (**b**) high-angle area expanded pattern; (**c**) lattice parameter *a*; (**d**) FWHM of (111) and (200) planes.

The XRD patterns of ZrN powders and TiN powders are shown in Figure 1. As expected, ZrN and TiN were the main crystallized phases for ZrN powders and TiN powders, respectively. In addition, a trace of ZrO_2_ impurity was detected. This was attributed to the presence of a small amount of ZrO_2_ in the initial ZrN powders. The XRD patterns of the sintered samples are shown in Figure 2. As shown in Figure 1, the (111) diffraction peak of ZrN powders was stronger than that of the (200) diffraction peak. However, for pure ZrN ceramic, the (111) diffraction peak was slightly lower than that of (200) diffraction peak. The results indicate that ZrN grains underwent oriented growth during the sintering process. This phenomenon was also reported in other ceramic composites, which could be attributed to the applied magnetic field and pressure [20,21,22]. As shown in Figure 2a, the (Zr, Ti)N solid solution was the main crystalline phase for ZT10, ZT20, and ZT30, demonstrating that Ti atoms were dissolved into the ZrN lattice during the sintering process. The effect of the TiN content on the lattice parameters of the (Zr, Ti)N solid solution composites is demonstrated by the enlarged view of (111) and (200) diffraction peaks and the calculated lattice parameters, as shown in Figure 2b and c, respectively. According to the expanded patterns in Figure 2b, with the higher TiN content, diffraction peaks of (Zr, Ti)N shifted to the higher angle. It is well-known that the atomic radius of Zr (∼1.62 Å) is larger than that of Ti (∼1.47 Å) [23]. Therefore, with higher TiN content, more and more Ti atoms dissoluted into the ZrN lattice, and the interplanar spacing of the (Zr, Ti)N solid solution gradually decreased. According to the Bragg equation [24] *nλ* = 2*d*sin*θ*, the Bragg angle (*θ*) increases with a decrease in interplanar spacing (*d*). Therefore, with higher TiN content, the diffraction peaks of (Zr, Ti)N shifted to the higher diffraction angle, as shown in Figure 2b. Therefore, the lattice parameter values of the (Zr, Ti)N phase decreased with higher TiN content, as tabulated in Figure 1c. It is well-known that the full width at half maximum (FWHM) of XRD peaks is closely related to the grain size. As shown in Figure 2d, the FWHM of (111) and (200) diffraction peaks decreased first and then increased with the increase in the TiN doping amount. The results indicate that the addition of TiN could promote grain refinement. 

### 3.2. Microstructure and Density

**Figure 3:**
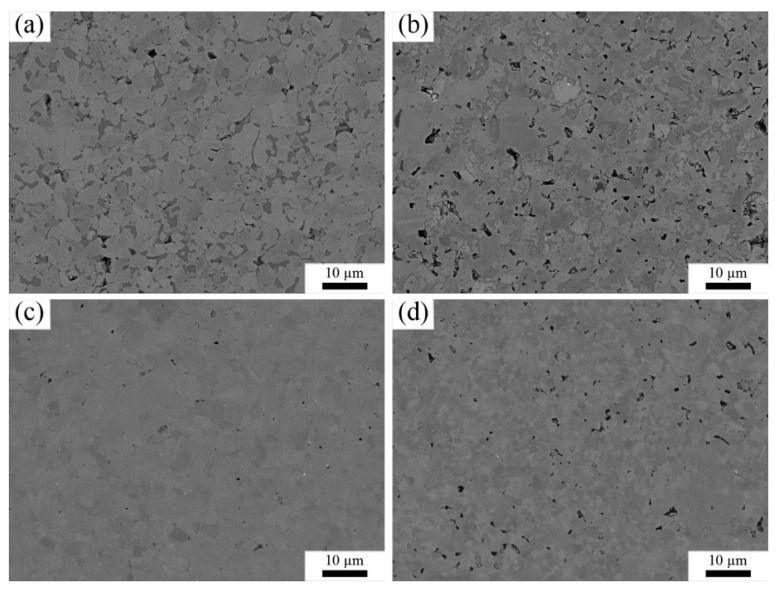
Backscattered electron (BSE) images from the polished surface of the sintered samples. (**a**) ZN, (**b**) ZT10, (**c**) ZT20, (**d**) ZT30.

**Figure 4:**
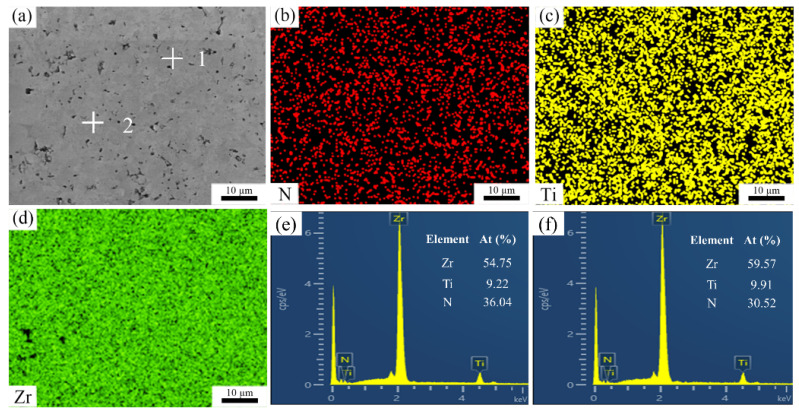
(**a**) BSE image of sample ZT10, (**b**)–(**d**) EDS mapping of N, Ti, and Zr, (**e**) and (**f**) EDS spectra of spots 1 and 2, respectively.

Figure 3 indicates BSE images of the polished surfaces of the sintered samples. It should be pointed out that although some pores were observed on the surfaces, these were caused by grinding and polishing because the Vickers hardness of the composite was far lower than that of the diamond. By grinding the surface of the samples with a diamond grinding disc, the ceramic substrate was scratched by diamond particles. There were no distinct phases in (Zr, Ti)N composites. In order to identify the microstructure in detail, the energy-dispersive spectrometer (EDS) spectra of sample ZT10 are illustrated in Figure 4. The results indicate that Zr and Ti elements were uniformly distributed, revealing the compositional homogeneity on the microscale. Furthermore, two different color areas of the polished surface were characterized in the EDS spectrum. As can be seen from Figure 4, Zr, Ti, and N elements with an atomic composition of 54.75:9.22:36.04 and 59.57:9.91:30.52 were presented in the zone of spots 1 and 2 (Figure 4a), respectively. The results of the point scan on two grains show similar elemental compositions. By combining the XRD and the EDS results, it can be inferred that the Ti atoms were dissolved into the ZrN lattice, and the (Zr, Ti)N solid solution was formed. 

**Figure 5:**
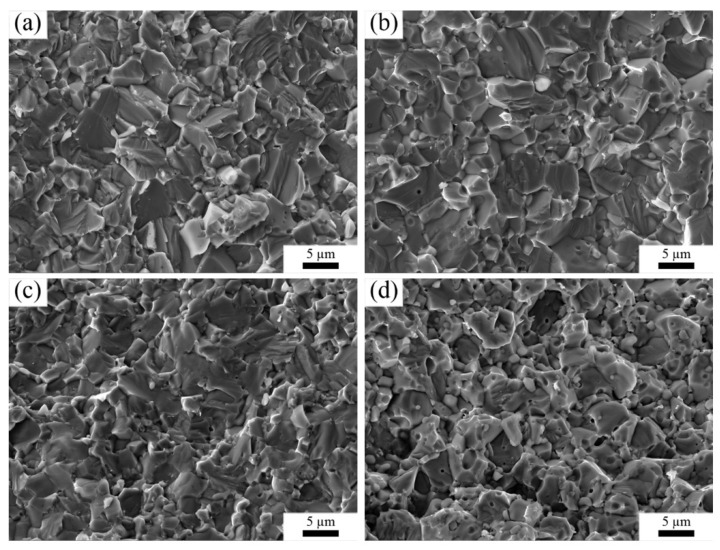
Scanning electron microscopy (SEM) images of the fractured surface of the sintered samples. (**a**) ZN, (**b**) ZT10, (**c**) ZT20, (**d**) ZT30.

Figure 5 shows the typical fracture surfaces of sintered samples. Notably, some tiny round pores were observed in all samples, which was mainly due to the rapid grain boundary migration caused by the high heating rate [25]. From the fracture morphologies of as-prepared ceramics, no pores can be found. The fracture surface of monolithic ZrN ceramic shows apparent smooth and cleavage surfaces, which indicates that intergranular and transgranular fracture modes coexist. As can be observed in Figure 5a,b, the morphology of the fracture surface of sample ZT10 was similar to that of sample ZN. For sample ZT20, most grains had smooth surfaces, indicating that the fracture mode was dominated by transgranular fracture. In Figure 5d, the morphology of the fracture surface was obviously changed. Furthermore, the fracture mode of ZT20 and ZT30 demonstrated a low bonding strength of the matrix skeleton, which was detrimental to the mechanical properties of the composites.

**Figure 6:**
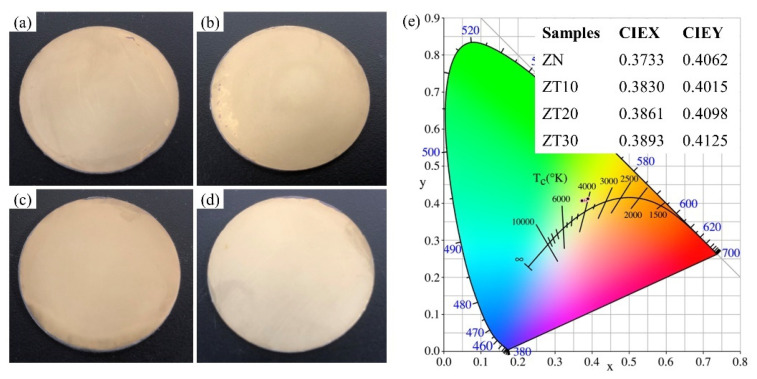
Pictures of the as-prepared ceramics: (**a**) ZN, (**b**) ZT10, (**c**) ZT20, (**d**) ZT30; (**e**) the corresponding color coordinates and color temperature diagram.

Figure 6 illustrates the pictures of various as-prepared ceramics and the corresponding emission color in the CIE chromaticity diagram. As shown in Figure 6, all samples exhibited similar optical properties. In order to accurately characterize the optical properties of the composites, the CIE chromaticity coordinates of as-prepared samples were measured. As shown in Figure 6e, the chromaticity coordinates of monolithic ZrN ceramic were (0.3733, 0.4062), which belonged to the yellow region. With an increase in TiN content, the chromaticity coordinates were (0.3830, 0.4015), (0.3861, 0.4098), and (0.3893, 0.4125) for samples ZT10, ZT20, and ZT30, respectively. The results indicate that the addition of TiN has no obvious influence on the optical properties of the composites. The (Zr, Ti)N composites have potential applications in jewelry fields, coating materials, and other fields.

### 3.3. Mechanical Properties

**Figure 7:**
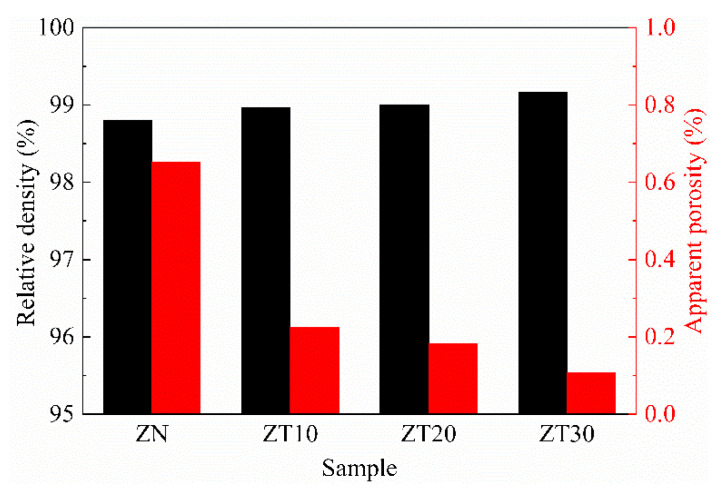
The relative density and apparent porosity of the sintered samples.

Figure 7 shows the relative density and apparent porosity of the as-prepared samples. As shown in Figure 7, the relative density of monolithic ZrN ceramic was 98.80%. The result indicates that ZrN ceramic could achieve high densification using SPS at 2000 ^o^C and 50 MPa with a holding time of 15 min. With the increasing TiN content, the relative density of the samples increased slightly to 98.96%, 99.01%, and 99.18% for samples ZT10, ZT20, and ZT30, respectively. Apparent porosity represents the ratio of open porosity volume to the total volume of a sample. With the increasing TiN content, the open porosity of the samples decreased slightly, which was consistent with the variation in relative density. The densification of (Zr, Ti)N ceramics was slightly promoted by the addition of TiN content. This was presumably ascribed to the solid solutions improving the surface diffusion and the mass transport processes by increasing defect populations [12,26,27]. Therefore, the (Zr, Ti)N composite ceramics were densified well. 

**Figure 8:**
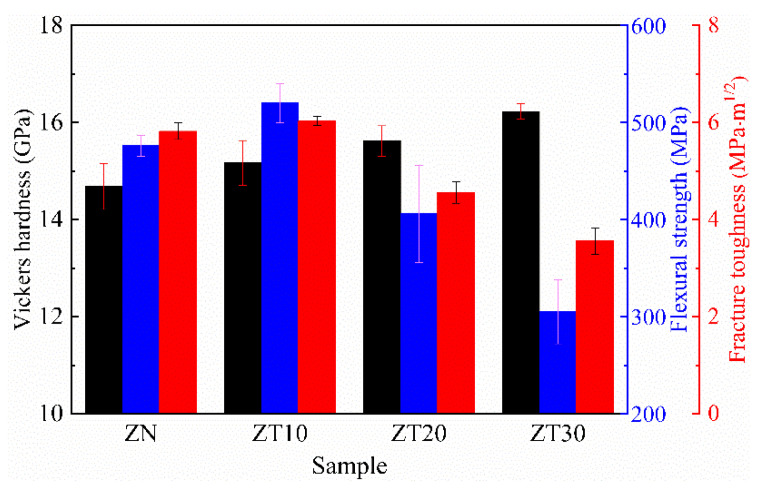
The mechanical properties of the sintered samples.

Figure 8 illustrates the Vickers hardness, flexural strength, and fracture toughness of the as-prepared samples. As shown in Figure 8, the Vickers hardness increased gradually with an increase in TiN content. This was mainly due to the intrinsic hardness of TiN (21 GPa) [28] is higher than that of ZrN (14.68 GPa). According to the mixture law, the addition of TiN to a ZrN matrix will lead to an improvement in the hardness of the composites. In this work, the flexural strength and fracture toughness of monolithic ZrN was 476 MPa and 5.82 MPa·m^1/2^, which was in good agreement with the results obtained by Tang et al. [4]. Nevertheless, TiN incorporation had a significant effect on flexural strength and fracture toughness. As shown in Figure 8, with the increment in TiN content, the flexural strength of composites was firstly enhanced and then significantly declined. Compared with monolithic ZrN ceramic, the flexural strength of ZT10 increased from 476 MPa to 520 MPa, which was mainly ascribed to solid solution strengthening [29]. However, with a further increase in the TiN addition, the flexural strength significantly decreased. It is well-known that flexural strength is dependent on microstructural features, such as grain size, orientation, and composition. As shown in Figure 5, the morphology of the fractured surface of the samples ZT20 and ZT30 was different from the samples ZN and ZT10. The fracture mode of ZT20 and ZT30 demonstrated a low bonding strength of the matrix skeleton. Therefore, the decreased flexural strength was mainly due to the decreasing grain bonding strength. 

**Figure 9:**
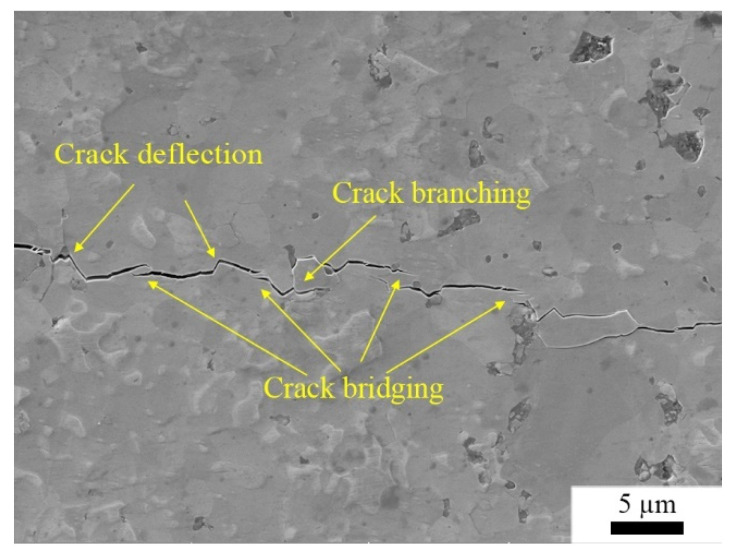
The crack propagation of sample ZT10.

Similar to the flexural strength trend, the fracture toughness of the composites slightly increased from 5.82 MPa·m^1/2^ to 6.06 MPa·m^1/2^ with the increase in TiN content up to 10 vol% and then significantly reduced to 4.56 MPa·m^1/2^ and 3.55 MPa·m^1/2^ for samples ZT20 and ZT30, respectively. It is well-known that flexural strength (*σ*) correlates to fracture toughness (*K_IC_*) through Equation (1) [30,31]:

(1) $$\;\sigma =\sqrt{\frac{\pi }{2}}\frac{K_{IC}}{\sqrt{d}}$$

where *d* is the diameter of the semi-circular surface flaw. Obviously, when *d* is a constant value, flexural strength is proportional to fracture toughness. In other words, flexural strength and fracture toughness have a similar tendency. This explains the variation in fracture toughness in Figure 8. We further explored the toughening mechanism of (Zr, Ti)N composite ceramics. Figure 9 shows the crack path of sample ZT10. As shown in Figure 9, multiple crack paths were present, i.e., crack deflection, crack branching, and crack bridging, which led to an increase in the length of the crack propagation path, thereby consuming more crack propagation energy and improving the fracture toughness. 

## 4. Conclusions

(Zr, Ti)N solid solution composites were fabricated by SPS of ZrN and TiN mixture powders, and the influence of the TiN addition on the sinterability, microstructure, color, and mechanical properties of ZrN ceramic was studied. The results indicate that the introduction of TiN had nearly no effect on the color performance of ZrN. With the increase in the TiN content, the relative density and Vickers hardness of the (Zr, Ti)N composites increased, while the flexural strength and fracture toughness were first enhanced and then declined. When the content of TiN was 10 vol%, the obtained (Zr, Ti)N ceramic had the best comprehensive mechanical properties: relative density 98.96%, Vickers hardness 15.17 GPa, flexural strength 520 MPa, and fracture toughness 6.03 MPa·m^1/2^. The fracture behavior of the (Zr, Ti)N composites underwent a mixed mode of transgranular fracture and intergranular fracture. The improved fracture toughness was attributed to the toughening mechanisms of the crack deflection, crack branching, and crack bridging.

## Data Availability

Not applicable.
